# Supplementary material for: A Likelihood Approach for Real-Time Calibration of Stochastic Compartmental Epidemic Models
Source: PLoS Comput Biol. 2017 Jan 17;13(1):e1005257. doi: 10.1371/journal.pcbi.1005257 (PMC5240920; doi:10.1371/journal.pcbi.1005257)
Supplement: S1 File — (TAR.GZ) [file pcbi.1005257.s014.tar.gz › HSPH_Online-SI-Revision/MSS10/n100-severe/n100-severe_NEW2_table0.pdf]

| | | | | | | | | | | | | | | | | | | | | | | | | | | | | | | | | | | | | | | | | |
|---|---|---|---|---|---|---|---|---|---|---|---|---|---|---|---|---|---|---|---|---|---|---|---|---|---|---|---|---|---|---|---|---|---|---|---|---|---|---|---|---|
| 1 | newly | 3 weeks | spec | 5129. | 3534.18 | {3380., 3708.} | 31.0942 | 0.162022 | newly | 3 weeks | cumul | 17951. | 13902.3 | {13481., 14329.} | 22.5543 | 0.111127 | newly | 5 weeks | spec | 3152. | 1789.09 | {1692., 1898.} | 43.2395 | 0.246456 | newly | 5 weeks | cumul | 25181. | 18243.5 | {17670., 18825.} | 27.5507 | 0.140079 | newly | 50 weeks | cumul | 33334. | 21864.2 | {21196., 22544.} | 34.4088 | 0.183263 |
| 2 | newly | 3 weeks | spec | 3271. | 3382.8 | {3178., 3601.} | 4.93427 | 0.0208073 | newly | 3 weeks | cumul | 16291. | 16516.5 | {15639., 17208.} | 2.95703 | 0.0126842 | newly | 5 weeks | spec | 952. | 1142.02 | {1047., 1235.} | 19.9601 | 0.0780011 | newly | 5 weeks | cumul | 19055. | 19667.8 | {18777., 20362.} | 3.74736 | 0.0158643 | newly | 57 weeks | cumul | 20150. | 21047.3 | {20271., 21689.} | 4.62695 | 0.0194943 |
| 3 | newly | 3 weeks | spec | 3370. | 3070.59 | {2964., 3191.} | 8.88457 | 0.0406143 | newly | 3 weeks | cumul | 19722. | 18553.6 | {18128., 18992.} | 5.9242 | 0.026589 | newly | 5 weeks | spec | 834. | 767.72 | {707., 829.} | 8.42446 | 0.0389059 | newly | 5 weeks | cumul | 22331. | 20868.5 | {20359., 21401.} | 6.54933 | 0.0294799 | newly | 58 weeks | cumul | 23238. | 21590.8 | {21072., 22022.} | 7.0883 | 0.0319918 |
| 4 | newly | 3 weeks | spec | 5397. | 3945.86 | {3258., 4483.} | 26.8879 | 0.138926 | newly | 3 weeks | cumul | 21357. | 17655.2 | {15693., 19119.} | 17.3332 | 0.083759 | newly | 5 weeks | spec | 2735. | 1539.73 | {1115., 1892.} | 43.7027 | 0.25718 | newly | 5 weeks | cumul | 27895. | 21708.8 | {18951., 23889.} | 22.1767 | 0.110495 | newly | 56 weeks | cumul | 32739. | 23923. | {20420., 26816.} | 26.9283 | 0.138479 |
| 5 | newly | 3 weeks | spec | 4803. | 3188.22 | {3005., 3328.} | 33.6202 | 0.178267 | newly | 3 weeks | cumul | 16297. | 12370.6 | {12022., 12766.} | 24.0928 | 0.119831 | newly | 5 weeks | spec | 3233. | 1705.75 | {1603., 1809.} | 47.2394 | 0.27815 | newly | 5 weeks | cumul | 23516. | 16439. | {15966., 16937.} | 30.0946 | 0.155597 | newly | 50 weeks | cumul | 34577. | 20269.7 | {19697., 20926.} | 41.3781 | 0.232052 |
| 6 | newly | 3 weeks | spec | 6383. | 4312.45 | {3911., 5114.} | 32.4385 | 0.172707 | newly | 3 weeks | cumul | 23683. | 19103.2 | {17951., 21045.} | 19.3381 | 0.0941654 | newly | 5 weeks | spec | 2924. | 1587.48 | {1342., 2090.} | 45.7086 | 0.272077 | newly | 5 weeks | cumul | 31005. | 23368.7 | {21750., 26530.} | 24.6293 | 0.124052 | newly | 56 weeks | cumul | 36697. | 25538.7 | {23723., 29648.} | 30.4067 | 0.159157 |
| 7 | newly | 3 weeks | spec | 5854. | 6505.76 | {6333., 6657.} | 11.1336 | 0.0457712 | newly | 3 weeks | cumul | 26186. | 27210.9 | {26828., 27562.} | 3.914 | 0.0166475 | newly | 5 weeks | spec | 2014. | 2630.78 | {2525., 2748.} | 30.6246 | 0.115812 | newly | 5 weeks | cumul | 31770. | 34082.4 | {33556., 34482.} | 7.27853 | 0.0304898 | newly | 57 weeks | cumul | 34576. | 37964.1 | {37475., 38385.} | 9.79902 | 0.0405789 |
| 8 | newly | 3 weeks | spec | 4856. | 3800.56 | {3603., 3973.} | 21.7348 | 0.106845 | newly | 3 weeks | cumul | 17949. | 15266.9 | {14640., 15859.} | 14.9432 | 0.0705159 | newly | 5 weeks | spec | 2677. | 1795.4 | {1680., 1900.} | 32.9324 | 0.173971 | newly | 5 weeks | cumul | 24358. | 19744.6 | {18902., 20355.} | 18.94 | 0.0913944 | newly | 53 weeks | cumul | 30079. | 23023.2 | {22317., 23689.} | 23.4576 | 0.116271 |
| 9 | newly | 3 weeks | spec | 4469. | 2132.28 | {1583., 2911.} | 52.2873 | 0.33517 | newly | 3 weeks | cumul | 27073. | 18309. | {15554., 21904.} | 32.3717 | 0.173495 | newly | 5 weeks | spec | 876. | 291.98 | {156., 499.} | 66.6689 | 0.524052 | newly | 5 weeks | cumul | 30073. | 19408.5 | {16380., 23399.} | 35.4621 | 0.194429 | newly | 58 weeks | cumul | 30570. | 19561.7 | {16479., 23681.} | 36.0101 | 0.198296 |
| 10 | newly | 3 weeks | spec | 5097. | 3607.48 | {3226., 4182.} | 29.2235 | 0.152365 | newly | 3 weeks | cumul | 20668. | 16912.3 | {15748., 18387.} | 18.1715 | 0.0878697 | newly | 5 weeks | spec | 2025. | 1278.3 | {1056., 1638.} | 36.8741 | 0.206136 | newly | 5 weeks | cumul | 25993. | 20386.1 | {18861., 22689.} | 21.5708 | 0.106642 | newly | 51 weeks | cumul | 29196. | 22092.8 | {20272., 24963.} | 24.3294 | 0.122608 |
| 11 | newly | 3 weeks | spec | 4623. | 2062.63 | {1887., 2254.} | 55.3833 | 0.351589 | newly | 3 weeks | cumul | 21770. | 14427.6 | {13358., 15248.} | 33.7271 | 0.179226 | newly | 5 weeks | spec | 1559. | 406.26 | {323., 505.} | 73.941 | 0.590402 | newly | 5 weeks | cumul | 26128. | 15767.2 | {14662., 16622.} | 39.6541 | 0.21984 | newly | 57 weeks | cumul | 28027. | 16064.5 | {14935., 16902.} | 42.6822 | 0.242192 |
| 12 | newly | 3 weeks | spec | 5357. | 3776.6 | {3632., 3934.} | 29.5016 | 0.152043 | newly | 3 weeks | cumul | 18503. | 15151.4 | {14725., 15561.} | 18.1136 | 0.086895 | newly | 5 weeks | spec | 3340. | 1814.43 | {1706., 1944.} | 45.6757 | 0.265563 | newly | 5 weeks | cumul | 26203. | 19634.4 | {19066., 20143.} | 25.0683 | 0.125441 | newly | 50 weeks | cumul | 35015. | 23053.1 | {22295., 23725.} | 34.1623 | 0.181642 |
| 13 | newly | 3 weeks | spec | 5099. | 4428.57 | {4294., 4528.} | 13.1483 | 0.0613223 | newly | 3 weeks | cumul | 17831. | 15744.8 | {15449., 16006.} | 11.6996 | 0.0540831 | newly | 5 weeks | spec | 3072. | 2741.26 | {2627., 2846.} | 10.7663 | 0.0496711 | newly | 5 weeks | cumul | 24961. | 22038.1 | {21624., 22440.} | 11.7099 | 0.0541343 | newly | 50 weeks | cumul | 33743. | 29777.3 | {29287., 30269.} | 11.7527 | 0.0543351 |
| 14 | newly | 3 weeks | spec | 4962. | 3484.76 | {3156., 3860.} | 29.7711 | 0.154601 | newly | 3 weeks | cumul | 15685. | 12821.6 | {11988., 13630.} | 18.2554 | 0.0880956 | newly | 5 weeks | spec | 3640. | 1967.6 | {1752., 2319.} | 45.9451 | 0.269382 | newly | 5 weeks | cumul | 23643. | 17450.2 | {16195., 18747.} | 26.1931 | 0.132594 | newly | 50 weeks | cumul | 36706. | 21964.3 | {20365., 24217.} | 40.1614 | 0.224124 |
| 15 | newly | 3 weeks | spec | 4360. | 4510.49 | {3954., 5232.} | 9.62638 | 0.0396353 | newly | 3 weeks | cumul | 17059. | 17275. | {15893., 19053.} | 6.17662 | 0.0261839 | newly | 5 weeks | spec | 2303. | 2213.22 | {1768., 2689.} | 16.3595 | 0.0723378 | newly | 5 weeks | cumul | 22470. | 22734.9 | {20414., 25447.} | 7.84611 | 0.0331359 | newly | 52 weeks | cumul | 26744. | 26811.2 | {23515., 30508.} | 10.1877 | 0.0431878 |
| 16 | newly | 3 weeks | spec | 5728. | 4632.32 | {4409., 4823.} | 19.1285 | 0.0924443 | newly | 3 weeks | cumul | 18259. | 16324.7 | {15765., 16812.} | 10.5935 | 0.0487694 | newly | 5 weeks | spec | 4036. | 2711.39 | {2581., 2850.} | 32.8199 | 0.173051 | newly | 5 weeks | cumul | 27272. | 22669.5 | {22035., 23315.} | 16.8762 | 0.0803885 | newly | 51 weeks | cumul | 38769. | 28951.5 | {28286., 29694.} | 25.323 | 0.126883 |
| 17 | newly | 3 weeks | spec | 4479. | 4880.56 | {4661., 5065.} | 8.97254 | 0.0370643 | newly | 3 weeks | cumul | 20396. | 20733.6 | {19855., 21509.} | 2.92469 | 0.0125101 | newly | 5 weeks | spec | 1516. | 1829.67 | {1682., 1962.} | 20.6906 | 0.0808429 | newly | 5 weeks | cumul | 24628. | 25637.6 | {24779., 26440.} | 4.20627 | 0.0177672 | newly | 57 weeks | cumul | 26798. | 28014.6 | {27172., 28770.} | 4.57889 | 0.0193403 |
| 18 | newly | 3 weeks | spec | 4189. | 4418.1 | {4247., 4591.} | 5.60516 | 0.0235359 | newly | 3 weeks | cumul | 17056. | 17495.2 | {17061., 17977.} | 2.82364 | 0.012036 | newly | 5 weeks | spec | 1958. | 2100.79 | {1993., 2214.} | 7.35291 | 0.0305191 | newly | 5 weeks | cumul | 22027. | 22709.6 | {22174., 23215.} | 3.26 | 0.0138785 | newly | 55 weeks | cumul | 25613. | 26526.3 | {25891., 27087.} | 3.59267 | 0.0152716 |
| 19 | newly | 3 weeks | spec | 3570. | 3318.23 | {3178., 3439.} | 7.05238 | 0.0319527 | newly | 3 weeks | cumul | 17052. | 16211.1 | {15794., 16647.} | 4.9349 | 0.0220664 | newly | 5 weeks | spec | 1298. | 1167.05 | {1073., 1251.} | 10.2288 | 0.0475639 | newly | 5 weeks | cumul | 20704. | 19386.1 | {18858., 19850.} | 6.36524 | 0.0286461 | newly | 55 weeks | cumul | 22668. | 20937.7 | {20376., 21467.} | 7.6331 | 0.0345701 |
| 20 | newly | 3 weeks | spec | 4499. | 3917.54 | {3713., 4111.} | 12.9242 | 0.06062 | newly | 3 weeks | cumul | 19401. | 17706.4 | {17046., 18314.} | 8.7346 | 0.0399192 | newly | 5 weeks | spec | 1849. | 1487.19 | {1306., 1621.} | 19.5679 | 0.096548 | newly | 5 weeks | cumul | 24272. | 21660.8 | {20890., 22441.} | 10.758 | 0.0497131 | newly | 53 weeks | cumul | 27031. | 23742. | {22761., 24578.} | 12.1675 | 0.0566966 |
| 21 | newly | 3 weeks | spec | 5237. | 4612.78 | {4171., 6131.} | 16.6951 | 0.07756 | newly | 3 weeks | cumul | 23703. | 22070.3 | {20968., 25367.} | 9.00654 | 0.0406921 | newly | 5 weeks | spec | 1742. | 1497.67 | {1255., 2457.} | 25.1429 | 0.116821 | newly | 5 weeks | cumul | 28600. | 26239.5 | {24678., 32129.} | 11.4675 | 0.0520695 | newly | 57 weeks | cumul | 30632. | 28007.5 | {26296., 35971.} | 12.9907 | 0.0588466 |
| 22 | newly | 3 weeks | spec | 5006. | 3378.93 | {3027., 3658.} | 32.5024 | 0.17328 | newly | 3 weeks | cumul | 21806. | 17612.7 | {16230., 18917.} | 19.23 | 0.0937086 | newly | 5 weeks | spec | 1880. | 923.2 | {731., 1090.} | 50.8936 | 0.316438 | newly | 5 weeks | cumul | 26703. | 20348.3 | {18797., 21603.} | 23.7977 | 0.119096 | newly | 55 weeks | cumul | 29097. | 21249.4 | {19361., 22487.} | 26.9706 | 0.137652 |
| 23 | newly | 3 weeks | spec | 1987. | 2198.7 | {2082., 2301.} | 10.6744 | 0.0436904 | newly | 3 weeks | cumul | 17807. | 18110.1 | {17412., 18613.} | 2.65609 | 0.0113852 | newly | 5 weeks | spec | 308. | 353.4 | {307., 410.} | 15.7792 | 0.061751 | newly | 5 weeks | cumul | 18940. | 19351.3 | {18624., 19908.} | 2.81679 | 0.0120326 | newly | 59 weeks | cumul | 19129. | 19567. | {18840., 20125.} | 2.86941 | 0.0122498 |
| 24 | newly | 3 weeks | spec | 4945. | 4699.37 | {4549., 4842.} | 4.97088 | 0.0222696 | newly | 3 weeks | cumul | 26504. | 25662.1 | {25118., 26130.} | 3.22872 | 0.0143056 | newly | 5 weeks | spec | 1389. | 1223.27 | {1132., 1296.} | 11.9316 | 0.0557158 | newly | 5 weeks | cumul | 30352. | 29320.4 | {28713., 29777.} | 3.39892 | 0.0150629 | newly | 60 weeks | cumul | 31658. | 30460.7 | {29832., 31004.} | 3.78214 | 0.0167828 |
| 25 | newly | 3 weeks | spec | 3439. | 2602.15 | {2293., 3183.} | 25.264 | 0.128683 | newly | 3 weeks | cumul | 21402. | 18642.3 | {17079., 20766.} | 13.4591 | 0.0634542 | newly | 5 weeks | spec | 698. | 470.98 | {330., 620.} | 34.8997 | 0.19445 | newly | 5 weeks | cumul | 23735. | 20237.9 | {18651., 22753.} | 15.3539 | 0.0731458 | newly | 60 weeks | cumul | 24404. | 20558.2 | {18960., 23215.} | 16.3924 | 0.0785367 |
| 26 | newly | 3 weeks | spec | 5656. | 3922.48 | {3520., 4518.} | 30.6492 | 0.161289 | newly | 3 weeks | cumul | 23609. | 19499.6 | {18358., 20988.} | 17.406 | 0.0836388 | newly | 5 weeks | spec | 2372. | 1273.07 | {1011., 1689.} | 46.3293 | 0.27914 | newly | 5 weeks | cumul | 29758. | 23045. | {21442., 25380.} | 22.5587 | 0.11203 | newly | 54 weeks | cumul | 33191. | 24540.6 | {22605., 27658.} | 26.0625 | 0.132545 |
| 27 | newly | 3 weeks | spec | 4510. | 4806.49 | {4505., 5047.} | 6.9306 | 0.0288496 | newly | 3 weeks | cumul | 21137. | 21603.3 | {20572., 22332.} | 3.21384 | 0.0137399 | newly | 5 weeks | spec | 1681. | 1766.94 | {1575., 1926.} | 7.74539 | 0.0323002 | newly | 5 weeks | cumul | 25596. | 26339.2 | {24917., 27273.} | 3.89819 | 0.016601 | newly | 55 weeks | cumul | 27946. | 28672.7 | {27066., 29605.} | 4.00544 | 0.0171013 |
| 28 | newly | 3 weeks | spec | 3624. | 3388.34 | {3172., 3612.} | 6.70806 | 0.0305624 | newly | 3 weeks | cumul | 14491. | 13569.6 | {12985., 14110.} | 6.35815 | 0.0287658 | newly | 5 weeks | spec | 2108. | 1642.43 | {1510., 1767.} | 22.0859 | 0.109132 | newly | 5 weeks | cumul | 19406. | 17608.3 | {16870., 18278.} | 9.26373 | 0.0424422 | newly | 54 weeks | cumul | 24332. | 20695.6 | {19926., 21433.} | 14.9451 | 0.0704832 |
| 29 | newly | 3 weeks | spec | 4912. | 4043.42 | {3891., 4198.} | 17.6828 | 0.0846838 | newly | 3 weeks | cumul | 20248. | 17999.5 | {17520., 18490.} | 11.1049 | 0.0512017 | newly | 5 weeks | spec | 2132. | 1595.14 | {1481., 1702.} | 25.1811 | 0.126587 | newly | 5 weeks | cumul | 25828. | 22175.1 | {21590., 22720.} | 14.1431 | 0.0662988 | newly | 55 weeks | cumul | 29513. | 24552.4 | {23917., 25147.} | 16.8081 | 0.0800052 |
| 30 | newly | 3 weeks | spec | 4348. | 3873.06 | {3680., 4057.} | 10.9232 | 0.050514 | newly | 3 weeks | cumul | 16097. | 15206.6 | {14621., 15754.} | 5.55923 | 0.0250161 | newly | 5 weeks | spec | 2202. | 1888.57 | {1773., 2000.} | 14.2339 | 0.067191 | newly | 5 weeks | cumul | 21394. | 19858.4 | {19036., 20490.} | 7.17776 | 0.0325047 | newly | 51 weeks | cumul | 26261. | 23353.8 | {22601., 24018.} | 11.0703 | 0.0510804 |
| 31 | newly | 3 weeks | spec | 1715. | 2022.27 | {1901., 2145.} | 17.9166 | 0.0710971 | newly | 3 weeks | cumul | 14771. | 16804.2 | {16173., 17475.} | 13.7648 | 0.0558127 | newly | 5 weeks | spec | 342. | 320.84 | {263., 381.} | 12.0702 | 0.0560875 | newly | 5 weeks | cumul | 15873. | 17935.9 | {17330., 18595.} | 12.996 | 0.0528775 | newly | 60 weeks | cumul | 16071. | 18146.6 | {17521., 18783.} | 12.915 | 0.0525699 |
| 32 | newly | 3 weeks | spec | 4733. | 4372.54 | {4233., 4530.} | 7.61589 | 0.0347329 | newly | 3 weeks | cumul | 23783. | 22690.9 | {22030., 23234.} | 4.62658 | 0.0206819 | newly | 5 weeks | spec | 1187. | 1232.88 | {1125., 1336.} | 7.18787 | 0.03083 | newly | 5 weeks | cumul | 27483. | 26289.1 | {25610., 27038.} | 4.36142 | 0.0194958 | newly | 58 weeks | cumul | 28771. | 27533.6 | {26929., 28301.} | 4.31229 | 0.0192832 |
| 33 | newly | 3 weeks | spec | 3999. | 3267.42 | {3103., 3413.} | 18.2941 | 0.0880198 | newly | 3 weeks | cumul | 20824. | 18390.1 | {17982., 18834.} | 11.6878 | 0.0540535 | newly | 5 weeks | spec | 1142. | 893.11 | {824., 968.} | 21.7942 | 0.107659 | newly | 5 weeks | cumul | 24133. | 21015.6 | {20451., 21513.} | 12.9177 | 0.060137 | newly | 58 weeks | cumul | 25686. | 21941.1 | {21451., 22499.} | 14.5797 | 0.0685043 |
| 34 | newly | 3 weeks | spec | 4878. | 3188.92 | {2977., 3367.} | 34.6265 | 0.185011 | newly | 3 weeks | cumul | 19185. | 15144.1 | {14440., 15837.} | 21.0627 | 0.103015 | newly | 5 weeks | spec | 2525. | 1121.88 | {1014., 1241.} | 55.5691 | 0.353657 | newly | 5 weeks | cumul | 25181. | 18198.7 | {17259., 18962.} | 27.7283 | 0.14128 | newly | 50 weeks | cumul | 30277. | 19686.7 | {18805., 20515.} | 34.978 | 0.187147 |
| 35 | newly | 3 weeks | spec | 3516. | 3234.84 | {3093., 3392.} | 7.99659 | 0.0365204 | newly | 3 weeks | cumul | 21757. | 21466.3 | {20812., 22093.} | 2.32247 | 0.0102166 | newly | 5 weeks | spec | 674. | 639.34 | {555., 721.} | 8.82789 | 0.0403263 | newly | 5 weeks | cumul | 24081. | 23573.6 | {22881., 24159.} | 2.59765 | 0.0114788 | newly | 59 weeks | cumul | 24720. | 24068.8 | {23369., 24672.} | 2.95922 | 0.0131059 |
| 36 | newly | 3 weeks | spec | 6059. | 7230.09 | {7006., 7341.} | 19.3281 | 0.0761915 | newly | 3 weeks | cumul | 27874. | 30229.8 | {29597., 30635.} | 8.45157 | 0.0350732 | newly | 5 weeks | spec | 2044. | 2715.8 | {2542., 2796.} | 32.8669 | 0.120932 | newly | 5 weeks | cumul | 33550. | 37496.8 | {36835., 37786.} | 11.7641 | 0.0479815 | newly | 57 weeks | cumul | 35952. | 41067.1 | {40243., 41375.} | 14.2276 | 0.0571358 |
| 37 | newly | 3 weeks | spec | 2491. | 2009.58 | {1838., 2166.} | 20.0329 | 0.0977905 | newly | 3 weeks | cumul | 16769. | 15240.8 | {13944., 16222.} | 9.50432 | 0.0439263 | newly | 5 weeks | spec | 544. | 340.01 | {268., 419.} | 37.9136 | 0.213609 | newly | 5 weeks | cumul | 18580. | 16420.1 | {15194., 17411.} | 11.9582 | 0.0558243 | newly | 59 weeks | cumul | 19084. | 16658.2 | {15450., 17587.} | 13.0147 | 0.0610739 |
| 38 | newly | 3 weeks | spec | 4614. | 3749.84 | {3598., 3914.} | 18.7291 | 0.0902705 | newly | 3 weeks | cumul | 23415. | 20408.1 | {19872., 20937.} | 12.8419 | 0.0597726 | newly | 5 weeks | spec | 1497. | 1048.84 | {964., 1126.} | 29.9372 | 0.155385 | newly | 5 weeks | cumul | 27574. | 23464.8 | {22884., 23990.} | 14.9025 | 0.0701544 | newly | 57 weeks | cumul | 29436. | 24548.8 | {24016., 25094.} | 16.6027 | 0.0789132 |
| 39 | newly | 3 weeks | spec | 3711. | 3474.58 | {3273., 3659.} | 6.44247 | 0.0293206 | newly | 3 weeks | cumul | 14691. | 14275.3 | {13637., 14884.} | 3.38779 | 0.0150835 | newly | 5 weeks | spec | 1875. | 1604.99 | {1489., 1743.} | 14.4005 | 0.0683037 | newly | 5 weeks | cumul | 19116. | 18282.1 | {17516., 19077.} | 4.49304 | 0.0201505 | newly | 54 weeks | cumul | 22836. | 21156.5 | {20336., 22000.} | 7.36477 | 0.0334394 |
| 40 | newly | 3 weeks | spec | 4019. | 3136.74 | {2900., 3348.} | 21.9522 | 0.108321 | newly | 3 weeks | cumul | 16800. | 15037.8 | {14110., 15900.} | 10.4893 | 0.0485478 | newly | 5 weeks | spec | 1644. | 1042.26 | {892., 1190.} | 36.6022 | 0.200427 | newly | 5 weeks | cumul | 20945. | 17932.1 | {16839., 18852.} | 14.3848 | 0.0678322 | newly | 57 weeks | cumul | 23590. | 19206. | {18133., 20179.} | 18.5843 | 0.08964 |
| 41 | newly | 3 weeks | spec | 3584. | 2869.63 | {2704., 3017.} | 19.9322 | 0.0969327 | newly | 3 weeks | cumul | 19699. | 17691.4 | {17108., 18191.} | 10.1913 | 0.0468305 | newly | 5 weeks | spec | 987. | 677.51 | {595., 767.} | 31.3566 | 0.165576 | newly | 5 weeks | cumul | 22587. | 19782.4 | {19183., 20376.} | 12.4168 | 0.0577126 | newly | 59 weeks | cumul | 23519. | 20385.4 | {19774., 20988.} | 13.3236 | 0.062228 |
| 42 | newly | 3 weeks | spec | 4951. | 7296.2 | {6819., 7782.} | 50.6795 | 0.179069 | newly | 3 weeks | cumul | 19337. | 24676.3 | {23900., 25660.} | 29.6176 | 0.112992 | newly | 5 weeks | spec | 2604. | 4710.54 | {4001., 5216.} | 87.0215 | 0.276504 | newly | 5 weeks | cumul | 25605. | 35373.8 | {33198., 37303.} | 40.987 | 0.149859 | newly | 52 weeks | cumul | 30663. | 49066.5 | {43195., 52859.} | 63.7263 | 0.214454 |
| 43 | newly | 3 weeks | spec | 3668. | 3429.22 | {3217., 3623.} | 6.7819 | 0.0308782 | newly | 3 weeks | cumul | 13660. | 12911.9 | {12309., 13459.} | 5.62006 | 0.0253346 | newly | 5 weeks | spec | 2047. | 1825.46 | {1685., 1958.} | 10.852 | 0.0506175 | newly | 5 weeks | cumul | 18398. | 17282.8 | {16624., 18100.} | 6.15485 | 0.0278261 | newly | 51 weeks | cumul | 23176. | 21216.6 | {20349., 22095.} | 8.45465 | 0.0386257 |
| 44 | newly | 3 weeks | spec | 5502. | 6454.87 | {6252., 6643.} | 17.3186 | 0.06917 | newly | 3 weeks | cumul | 26971. | 29288.7 | {28705., 29885.} | 8.59342 | 0.0357373 | newly | 5 weeks | spec | 1807. | 2172.79 | {2035., 2280.} | 20.2429 | 0.0793785 | newly | 5 weeks | cumul | 31936. | 35293.6 | {34633., 35756.} | 10.5136 | 0.0433365 | newly | 58 weeks | cumul | 33949. | 37858.6 | {37228., 38203.} | 11.5161 | 0.04725 |
| 45 | newly | 3 weeks | spec | 5745. | 4394.9 | {4110., 4787.} | 23.5004 | 0.117126 | newly | 3 weeks | cumul | 23088. | 19863.9 | {19070., 20884.} | 13.9644 | 0.065589 | newly | 5 weeks | spec | 2549. | 1577.29 | {1384., 1871.} | 38.1212 | 0.211488 | newly | 5 weeks | cumul | 29573. | 24132. | {23088., 25567.} | 18.3986 | 0.0886576 | newly | 56 weeks | cumul | 33764. | 26172.2 | {25035., 28374.} | 22.4848 | 0.111117 |
| 46 | newly | 3 weeks | spec | 5945. | 4640.99 | {3994., 5566.} | 21.9346 | 0.111559 | newly | 3 weeks | cumul | 25387. | 21655.9 | {19946., 23863.} | 14.697 | 0.0702713 | newly | 5 weeks | spec | 2372. | 1558.89 | {1157., 2111.} | 34.2795 | 0.195142 | newly | 5 weeks | cumul | 31718. | 25977. | {23477., 29546.} | 18.1 | 0.0887163 | newly | 55 weeks | cumul | 35057. | 27860.8 | {24951., 32301.} | 20.5272 | 0.102441 |
| 47 | newly | 3 weeks | spec | 4772. | 3298.4 | {3140., 3455.} | 30.8801 | 0.16069 | newly | 3 weeks | cumul | 17003. | 12849.8 | {12489., 13289.} | 24.4265 | 0.121757 | newly | 5 weeks | spec | 2927. | 1741.91 | {1627., 1845.} | 40.4882 | 0.225971 | newly | 5 weeks | cumul | 23808. | 17006.6 | {16438., 17549.} | 28.5678 | 0.146237 | newly | 51 weeks | cumul | 31769. | 20844.6 | {20110., 21443.} | 34.3869 | 0.183146 |
| 48 | newly | 3 weeks | spec | 5735. | 4453.01 | {3599., 4943.} | 22.3538 | 0.112877 | newly | 3 weeks | cumul | 21931. | 19067.2 | {17006., 20362.} | 13.0583 | 0.0617552 | newly | 5 weeks | spec | 2847. | 1823.22 | {1321., 2137.} | 35.96 | 0.201158 | newly | 5 weeks | cumul | 29108. | 23806.7 | {20548., 25796.} | 18.2124 | 0.0888746 | newly | 56 weeks | cumul | 34186. | 26563.8 | {22153., 29076.} | 22.2963 | 0.111832 |
| 49 | newly | 3 weeks | spec | 3521. | 2644.44 | {2514., 2784.} | 24.8952 | 0.124805 | newly | 3 weeks | cumul | 23225. | 20520. | {19753., 21245.} | 11.6471 | 0.0539687 | newly | 5 weeks | spec | 685. | 428.65 | {370., 498.} | 37.4234 | 0.206503 | newly | 5 weeks | cumul | 25469. | 22032. | {21324., 22756.} | 13.4948 | 0.0631301 | newly | 60 weeks | cumul | 25996. | 22308.7 | {21572., 23060.} | 14.1842 | 0.0666013 |
| 50 | newly | 3 weeks | spec | 2974. | 2906.5 | {2727., 3054.} | 3.74781 | 0.0167032 | newly | 3 weeks | cumul | 18646. | 18035.6 | {17306., 18612.} | 3.52939 | 0.0157162 | newly | 5 weeks | spec | 685. | 673.64 | {613., 745.} | 6.9927 | 0.0308811 | newly | 5 weeks | cumul | 20804. | 20132.2 | {19386., 20754.} | 3.4528 | 0.0153652 | newly | 59 weeks | cumul | 21381. | 20718.4 | {20009., 21336.} | 3.31154 | 0.0147222 |
